# The impact of EGFR T790M mutation status following the development of Osimertinib resistance on the efficacy of Osimertinib in non‐small cell lung cancer: A meta‐analysis

**DOI:** 10.1111/crj.13748

**Published:** 2024-04-07

**Authors:** Liuxian Guo, Guojin Zhou, Min Huang, Kejing Tang, Jing Xu, Jie Chen

**Affiliations:** ^1^ Department of Pharmacy, The First Affiliated Hospital Sun Yat‐sen University Guangzhou China; ^2^ School of Pharmaceutical Sciences, Sun Yat‐Sen University E‐132# Waihuandong Road Guangzhou University City Guangzhou China; ^3^ Department of Pharmacy, Dermatology Hospital Southern Medical University Guangzhou China

**Keywords:** drug resistance, EGFR T790M mutation, meta‐analysis, non‐small cell lung cancer, Osimertinib

## Abstract

**Background:**

Previous studies have suggested that loss of the EGFR T790M gene mutation may contribute to the development of resistance to Osimertinib in non‐small cell lung cancer (NSCLC).

**Aims:**

This study aims to assess the relationship between the clinical effectiveness of Osimertinib in NSCLC patients and the T790M mutation status following resistance to Osimertinib and examine differences between plasma and tissue tests and between Asian and non‐Asian groups.

**Methods:**

The PubMed, Web of Science, Cochrane, and EMBASE databases were comprehensively searched for studies on the association between T790M mutation status and the efficacy of Osimertinib between January 2014 and November 2023. Meta‐analysis was carried out using Review Manager 5.4 software.

**Results:**

After evaluating 2727 articles, a total of 14 studies were included in the final analysis. Positive correlations between EGFR T790M mutation status after Osimertinib resistance and longer PFS (HR: 0.44, 95% CI: 0.30–0.66), longer OS (HR: 0.3, 95% CI: 0.10–0.86), longer TTD (HR: 0.69, 95% CI: 0.45–1.07), and improved clinical outcomes including PFS and TTD subgroups (HR: 0.58, 95% CI: 0.47–0.73) were observed. Subgroup analysis revealed that, compared with the blood tests, the results of the T790M mutation tests by the tissue are more significant (HR: 0.24, 95% CI: 0.11–0.52 for tissue tests; HR: 0.47, 95% CI: 0.22–1.00 for plasma tests), and the PFS of Osimertinib were similar for Asian and non‐Asian patients (HR: 0.46, 95% CI: 0.31–0.68 for Asians; HR: 0.12, 95% CI: 0.01–1.27 for non‐Asians).

**Conclusions:**

Persistence of the T790M gene mutation after the development of Osimertinib resistance is associated with higher therapeutic benefits of Osimertinib in NSCLC patients. The results of tissue detection are more significant than those of plasma detection.

## Graphical abstract

Persistence of the T790M gene mutation after the development of Osimertinib resistance is associated with higher therapeutic benefits of Osimertinib in NSCLC patients. The results of tissue detection are more significant than those of plasma detection. Furthermore, Asian patients were found to experience similar benefits from Osimertinib treatment as non‐Asian patients.

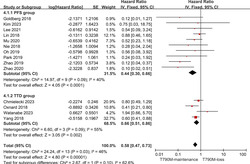

## 1 INTRODUCTION

Lung cancer is the second most common cancer worldwide and the main cause of cancer death, according to a recent GLOBOCAN report.^1^ Non‐small cell lung cancer (NSCLC) accounts for approximately 85% of all lung cancer cases.^2^ The treatment landscape for NSCLC has shifted with the emergence of tyrosine kinase inhibitors (TKIs). Because not every NSCLC patient benefits from first‐ and second‐generation EGFR‐TKIs due to the development of T790M mutation‐mediated resistance, a third‐generation EGFR‐TKI, namely, Osimertinib, has been developed.

Osimertinib targets the EGFR T790M mutation, irreversibly binds to the cysteine‐797 residue through covalent linkage, and exhibits highly selective inhibitory activity.^3^ Compared with standard EGFR‐TKIs, Osimertinib performed better progression‐free survival (PFS) than comparator EGFR‐TKIs (18.9 months vs. 10.2 months) in the FLAURA trial involving patients with previously untreated EGFR‐mutated advanced NSCLC.^4^, ^5^ Furthermore, Osimertinib has been recommended for EGFR sensitizing (19del/L858R) and EGFR T790M mutation‐positive advanced NSCLC in the 2023 National Comprehensive Cancer Network guidelines,^6^ as well as in the European Society of Medical Oncology (ESMO),^7^ the Chinese Society of Clinical Oncology (CSCO),^8^, ^9^ and the Chinese Medical Association.^10^

Resistance to second‐line Osimertinib is inevitable after a prolonged period of use. Two types of resistance mechanisms have been confirmed: (i) EGFR‐dependent resistance, which includes mutations such as the EGFR C797 mutation, EGFR L718 mutation, and EGFR gene amplification; and (ii) EGFR‐independent resistance, which includes mutations such as alterations in MET, HER2, and KRAS.^11^ Among them, the loss of the T790M mutation occurs in 45% of cases.^12^ So these diverse resistance mechanisms can essentially be divided into two groups depending on whether the T790M mutation is lost or maintained. A meta‐analysis study by Zhao et al.^13^ in 2020 showed that the persistence of the T790M mutation was associated with the development of resistance to Osimertinib. However, due to the limited papers examined in that earlier meta‐analysis study, these findings remain controversial in real‐world applications. For example, a prospective study by Lee et al.^14^ demonstrated that persistence of the T790M mutation was a potential prognostic factor of Osimertinib, whereas a retrospective analysis by Mu et al.^15^ revealed that PFS was unaffected by the loss or maintenance of the T790M mutation.

Studies have shown a direct relationship between acquired EGFR mutations and drug resistance in lung cancer patients treated with EGFR‐TKIs.^16^ Different detection methods and ethnic disparities are also closely interconnected. Genome‐wide association studies of NSCLC patients have revealed notable disparities between the germline susceptibility loci patterns and general background genetic structure of Asian and non‐Asian individuals.^17^ Thus, it is crucial to estimate the clinical efficacy of drugs by evaluating tumor characteristics and genetic changes during disease progression to properly guide clinical therapy.^18^, ^19^, ^20^

The aim of this study was to assess the relationship between the clinical effectiveness, including PFS, overall survival (OS), and time to discontinuation (TTD), in patients with non‐small cell lung cancer and the status of the T790M mutation following resistance to Osimertinib. Additionally, the study aimed to examine the differences between plasma and tissue test and between Asian and non‐Asian groups further.

## 2 METHODS

### 2.1 Search strategy

This work was carried out according to the Preferred Reporting Items for Systematic Reviews and Meta‐Analyses (PRISMA) guidelines.^21^ The study was registered in advance on the PROSPERO website under the registration number CRD42023413655. The PubMed, Web of Science, Cochrane, and EMBASE databases were searched for eligible English‐language articles published between January 2014 and November 2023. The search keywords included “NSCLC OR non‐small cell lung cancer OR adenocarcinoma,” “T790M,” “EGFR OR epidermal growth factor receptor,” “Osimertinib OR AZD9291 mesylate OR Tagrisso,” and “drug resistance.” Subject words and free words were used for permutation and combination retrieval. Details on the search strategies can be found in the Supporting Information.

### 2.2 Inclusion and exclusion criteria

The inclusion criteria for this study were as follows: (i) patients with NSCLC who were treated with Osimertinib and who eventually became resistant to Osimertinib; (ii) detailed survival data were reported in the literature, including PFS, OS, and TTD, and hazard ratios (HRs); and (iii) the baseline was T790M‐positive, and two genetic tests were conducted before and after the usage of Osimertinib to identify the mutation status of T790M and other genes.

The following articles were excluded: (i) articles with incomplete survival data and genetic testing times; (ii) combined use with other antineoplastic agents; (iii) duplicate articles; (iv) cell and animal experiments; and (v) case reports, letters, meeting abstracts, comments, editorials, and personal correspondence were excluded.

### 2.3 Data extraction

Two investigators independently extracted the following data: first author, year, study period, study type, region, mode of detection, number of patients who maintained or lost the T790M mutation, and individual survival outcomes, including PFS, OS, and TTD. PFS was determined by counting the number of days that had passed after the beginning of Osimertinib therapy up until either progressive disease (PD), death from any cause, or the last follow‐up date. The duration of OS was calculated as the interval between the start of Osimertinib treatment and the date of death or last follow‐up. TTD was calculated as the time until the end of therapy for any reason. Hazard ratios (HRs) were used as a measure for comparison. It was extracted from each published cohort of the original text or extracted from the Kaplan–Meier and Swimmer plot, by using the Engauge Digitizer 11.1 software previously described by Tierney et al. if survival data were not provided explicitly.^22^

### 2.4 Assessing risk of bias

The Newcastle–Ottawa scale (NOS) was used to independently evaluate the quality of the included studies.^23^, ^24^ Publication bias was evaluated using funnel plots generated by Review Manager 5.4 software. Substantially symmetrical funnel plots indicated that there was no publication bias or other bias present in the study. Incomplete funnel plots, for example, when the horn of the funnel plot was missing, were observed in cases of publication bias, such as, when certain negative outcomes were not published.

### 2.5 Statistical analysis

This study used HRs and corresponding 95% confidence intervals (95% CIs) to estimate time‐to‐event outcomes. Cox regression models were used to calculate the HRs and corresponding 95% CIs for each included study in cases where the data could not be directly retrieved. The *I* ^2^ index was used to determine the degree of heterogeneity. The fixed effects model was used for calculations when the *I* ^2^ index was <50% and the *p* value was <0.05, indicating that the degree of heterogeneity was minimal; otherwise, a random effects model was applied. All statistical analyses were calculated and analyzed using Review Manager 5.4 software.

## 3 RESULTS

### 3.1 Literature screening

**FIGURE 1:**
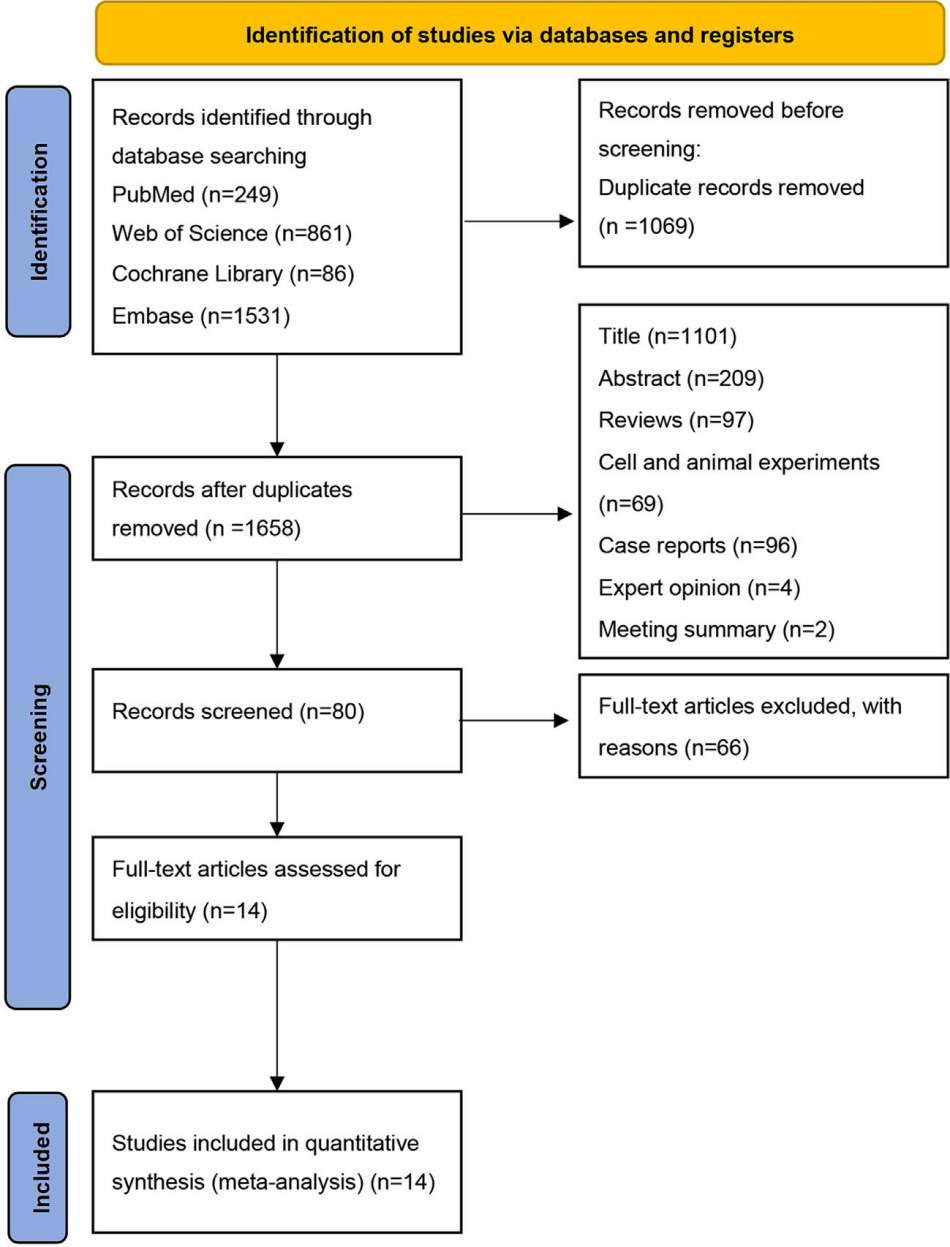
Flow diagram of the study selection process.

A total of 2727 articles published between January 2014 and November 2023 were retrieved. After screening, 14 articles consisting of 478 patients were selected for meta‐analysis. A flow diagram^25^ of the information collected in the studies is shown in Figure 1.

### 3.2 Data extraction and quality assessment

Patient characteristics, as well as the corresponding survival statistics, are shown in Table 1. In addition, the NOS scale was used to assess the quality of the publications. In general, the scores of the included articles ranged from 5 to 8, demonstrating that they were of moderate or good quality (Table 2).

**TABLE 1:** Characteristics of the studies included in this meta‐analysis.

Study	Study period	Type	Region	Total number of people	Patients		Mode of detection	Outcome	Pos/Neg HR	95% CIs
					T790M maintained	T790M lost				
Goldberg 2018^26^	Unknown	Retrospective Single‐center	Non‐Asian (America)	9	7	2	Tissue or plasma	PFS	0.118	0.011–1.327
Kim 2023^27^	June 2014–November 2018	Retrospective Single‐center	Asia (Korea)	23	9	14	Tissue or plasma	PFS	0.75	0.03–17.96
								OS	0.03	0–73.4
Lee 2021^14^	Unknown ‐ July 31, 2019	Prospective Single‐center	Asia (Korea)	29	7	22	Tissue	PFS	0.54	0.09–3.17
								OS	0.51	0.05–5.21
Lin 2018^28^	July 31, 2013 ‐ December 31, 2016	Prospective Single‐center	Asia (China)	41	18	23	Tissue or plasma	PFS	0.8771	0.465–1.652
Mu 2020^15^	March 1, 2017 ‐ December 31, 2018	Retrospective Single‐center	Asia (China)	49	24	25	Plasma	PFS	0.52	0.23–1.15
Nie 2018^29^	Unknown	Retrospective Single‐center	Asia (China)	9	7	2	Tissue	PFS	0.282	0.039–2.027
Oh 2019^30^	February 2016–June 2017	Retrospective Single‐center	Asia (Korea)	10	3	7	Tissue	PFS	0.56	0.08–3.89
Park 2019^31^	September 2016–September 2017	Prospective Single‐center	Asia (Korea)	7	3	4	Plasma	PFS	0.24	0.03–2.20
Zhao 2019^32^	January 2014–December 2016	Retrospective Single‐center	Asia (China)	31	16	15	Tissue	PFS	0.12	0.039–0.368
								OS	0.274	0.083–0.9
Zhao 2020^13^	Unknown	Retrospective Single‐center	Asia (China)	14	7	7	Tissue or plasma	PFS	0.098	0.019–0.513
Chmielecki 2023^5^	August 4, 2014 ‐ March 15, 2019	Prospective Multiple‐center	Asia and non‐Asian	68	29	39	Plasma	TTD	0.796	0.491–1.29
Oxnard 2018^33^	Unknown ‐ November 9, 2017	Retrospective Multiple‐center	Non‐Asian (America)	41	13	28	Plasma	TTD	0.411	0.21–0.804
Watanabe 2023^34^	December 2016–December 2019	Prospective Multiple‐center	Asia (Japan)	37	13	24	Plasma	TTD	1.94	0.66–5.74
Yang 2018^35^	May 14, 2014 ‐ November 1, 2015	Retrospective Multiple‐center	Asia and non‐Asian	110	58	52	Tissue	TTD	0.597	0.406–0.878

**TABLE 2:** Quality assessment of the studies used in this meta‐analysis based on the NOS scores.

	Selection					Outcome assessment			
Study	1	2	3	4	Comparability	1	2	3	Score
Goldberg 2018^26^	*		*	*	*	*	*	*	7
Kim 2023^27^	*	*	*	*	*	*	*	*	8
Lee 2021^14^	*		*	*	*	*	*		6
Lin 2018^28^	*		*	*	*	*	*	*	7
Mu 2020^15^	*	*	*	*	*	*	*	*	8
Nie 2018^29^	*		*	*		*	*	*	6
Oh 2019^30^	*		*	*	*	*	*		6
Park 2019^31^	*		*	*	*	*	*	*	7
Zhao 2019^32^	*		*	*	*	*	*		6
Zhao 2020^13^	*		*	*	*	*	*	*	7
Chmielecki 2023^5^	*	*		*	*	*	*	*	7
Oxnard 2018^33^	*		*	*	*	*			5
Watanabe 2023^34^	*	*	*	*	*	*	*	*	8
Yang 2018^35^	*	*	*	*		*	*	*	7

*Note*: * represents one point.

### 3.3 Meta‐analysis

#### 3.3.1 PFS

**FIGURE 2:**
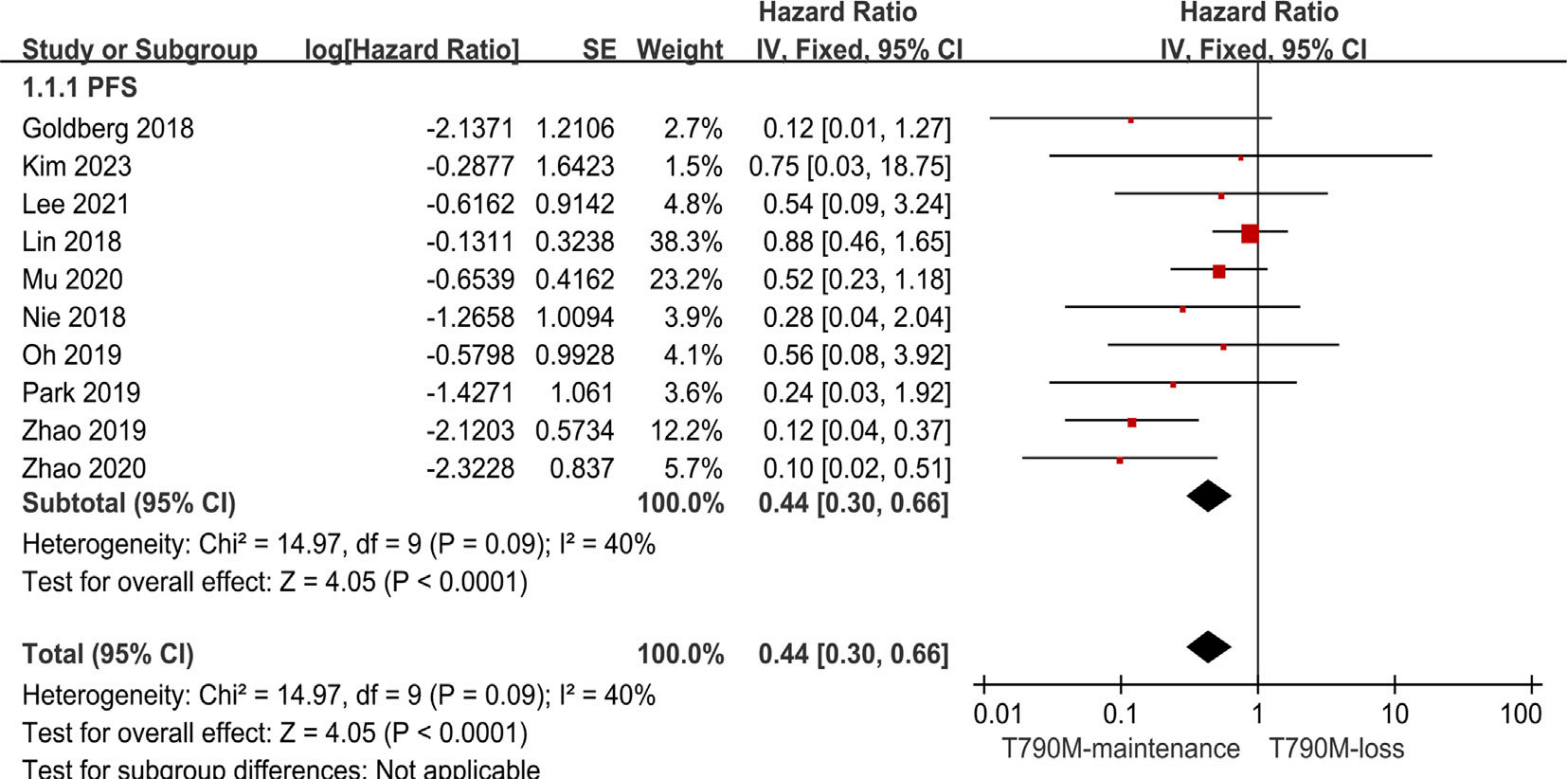
Forest plot of the PFS of T790M‐maintenance NSCLC patients compared with the T790M‐loss group following acquisition of resistance to Osimertinib and with a positive T790M mutation status at the baseline.

A total of 222 patients were selected from ten articles.^13^, ^14^, ^15^, ^26^, ^27^, ^28^, ^29^, ^30^, ^31^, ^32^ Of these 222 patients, 121 patients exhibited the loss of the T790M mutation subsequent to the emergence of resistance to Osimertinib (T790M‐loss group) and 101 patients maintained the T790M mutation (T790M‐maintenance group) while developing resistance to Osimertinib. Pooled analysis showed that the risk value for the T790M‐maintenance group was lower than for the T790M‐loss group (HR: 0.44, 95% CI: 0.30–0.66; Figure 2). Because the *I* ^2^ index was found to be <50% indicating that the heterogeneity was low among the 10 studies, a fixed effects model was used for fitting. The results suggested that the risk of T790M loss following resistance was greater than that of T790M maintenance, in other words, maintenance of the T790M mutation was associated with better PFS of Osimertinib in NSCLC patients.

#### 3.3.2 OS

**FIGURE 3:**
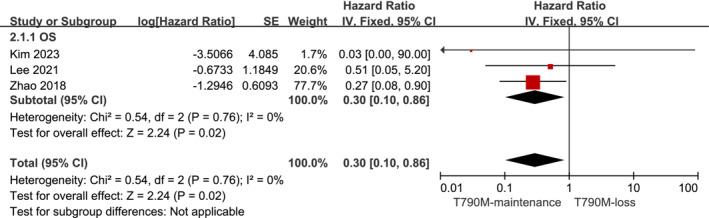
Forest plot of the OS of T790M‐maintenance NSCLC patients compared with the T790M‐loss group following acquisition of resistance to Osimertinib and with a positive T790M mutation status at the baseline.

Three of the ten studies^14^, ^27^, ^32^ provided OS data in addition to PFS data. The aggregated analysis reported a longer OS in the T790M‐maintenance group compared with the T790M‐loss group (HR: 0.3, 95% CI: 0.1–0.86; Figure 3) without heterogeneity (*I* ^2^ = 0).

#### 3.3.3 TTD

**FIGURE 4:**
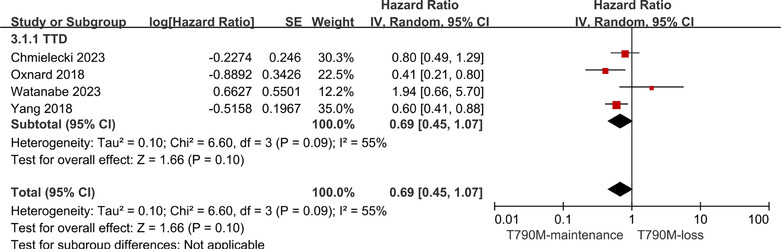
Forest plot of the TTD of T790M‐maintenance NSCLC patients compared with the T790M‐loss group following acquisition of resistance to Osimertinib and with a positive T790M mutation status at the baseline.

The other four articles^5^, ^33^, ^34^, ^35^ reported TTD data. Pooled analysis revealed that the T790M‐maintenance group had a longer TTD than the T790M‐loss group (HR: 0.69, 95% CI: 0.45–1.07; Figure 4) and that the *I* ^2^ index among them was found to be >50%; a random effects model was used for fitting.

#### 3.3.4 Combined survival data

**FIGURE 5:**
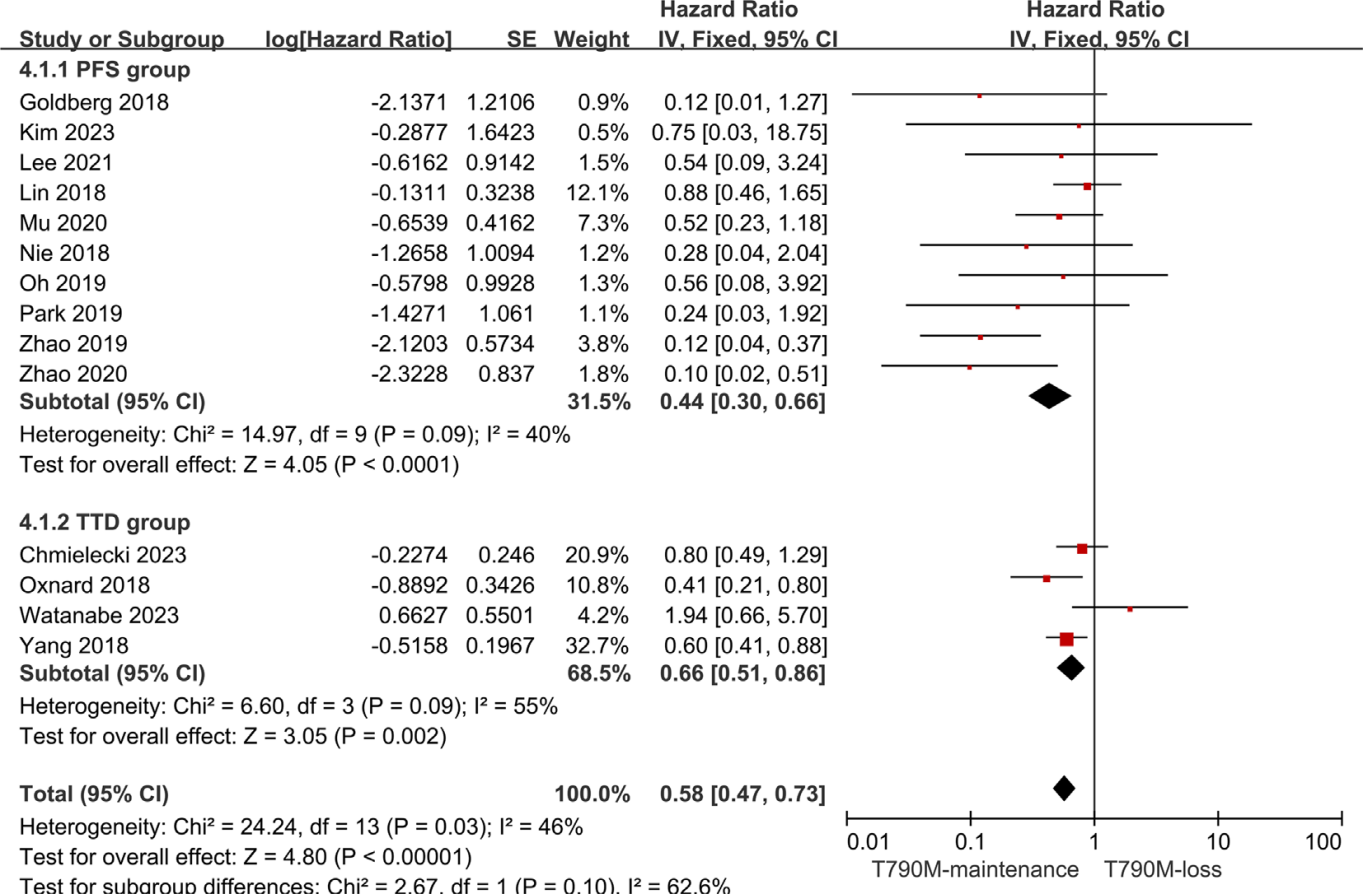
Forest plot of the survival outcomes including PFS and TTD in T790M‐maintenance NSCLC patients compared with the T790M‐loss group following acquisition of resistance to Osimertinib and with a positive T790M mutation status at the baseline.

Because PFS and TTD are all cancer patient survival outcomes, a total of 478 patients selected from 14 studies^5^, ^13^, ^14^, ^15^, ^26^, ^27^, ^28^, ^29^, ^30^, ^31^, ^32^, ^33^, ^34^, ^35^ were included in the subgroup analyses of survival outcomes. Results showed that the T790M‐maintenance group had longer survival outcomes than the T790M‐loss group (HR: 0.58, 95% CI: 0.47–0.73; Figure 5). In addition, the *I* ^2^ index was <50%, indicating that these results were credible. Thus, our findings confirmed that NSCLC patients who maintained their T790M mutation after developing Osimertinib resistance had better clinical outcomes than patients who lost the T790M mutation.

#### 3.3.5 Subgroup analysis

**FIGURE 6:**
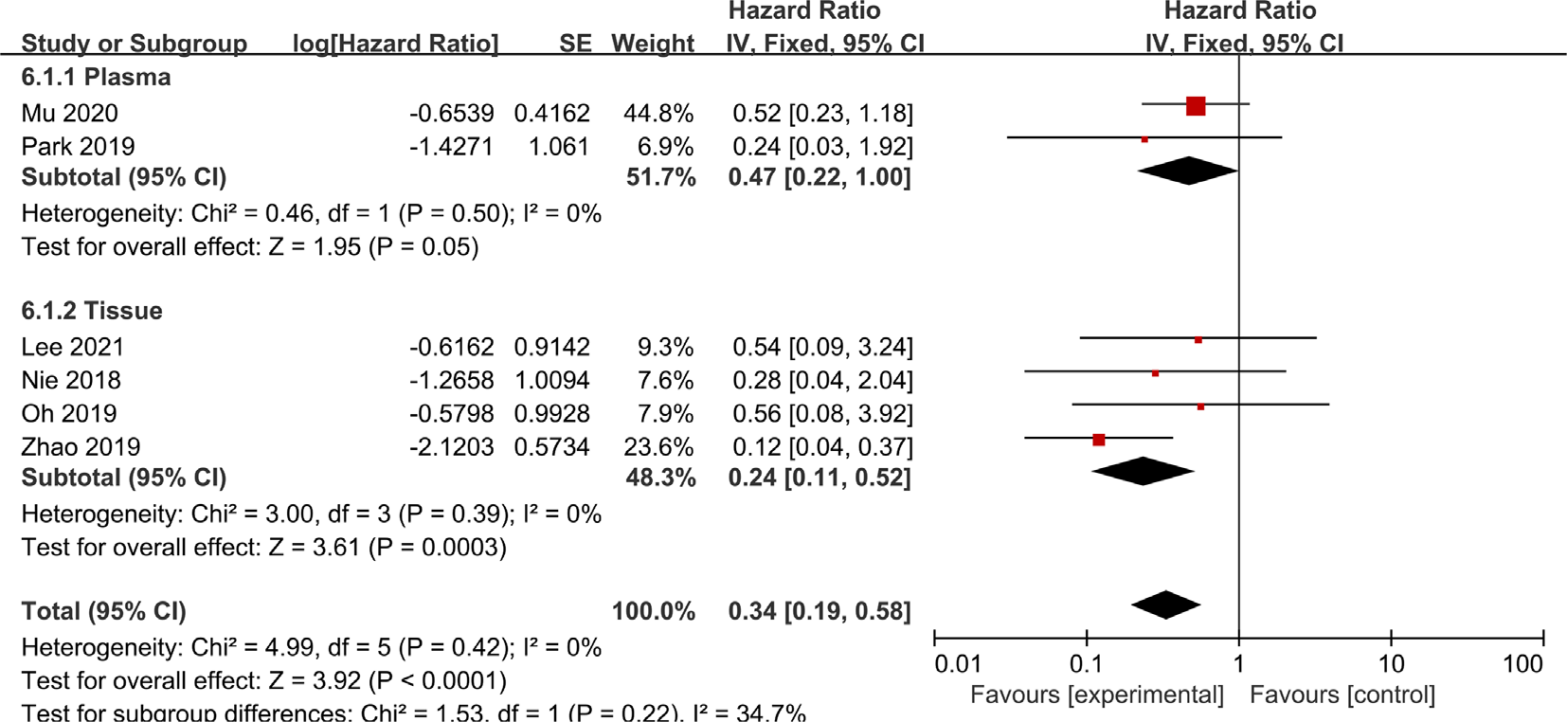
Subgroup analysis based on sample types. Differences in T790M mutation status and clinical efficacy were compared between plasma and tissue samples.

Due to the potential influence of sample types and detection techniques on the detection results of T790M mutation, we performed additional subgroup analysis based on the different sample types. Mu et al. and Park et al.^15^, ^31^ collected plasma samples for detection, whereas some articles^14^, ^29^, ^30^, ^32^ collected tissue samples. Other documents were excluded from the analysis due to the lack of PFS data for both tissue and plasma groups. According to the findings from the subgroup analysis, it was observed that the HR of the T790M‐maintenance group versus the T790M‐loss group, when using tissue samples for T790M mutation detection, was comparatively lower than that when using plasma samples (HR: 0.24, 95% CI: 0.11–0.52 for tissue samples; HR: 0.47, 95% CI: 0.22–1.00 for plasma samples; Figure 6).

**FIGURE 7:**
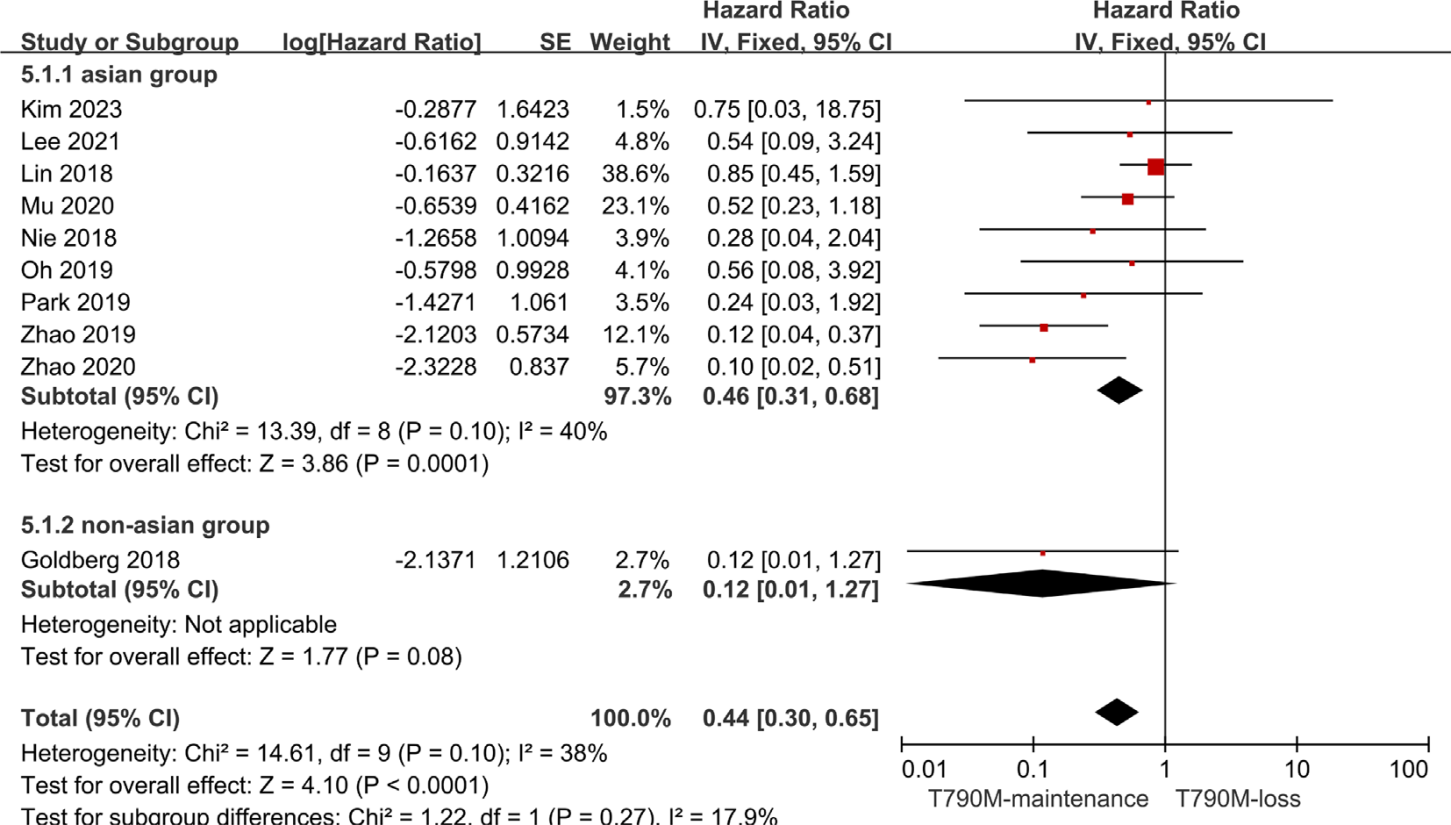
Subgroup analysis based on ethnicity. Differences in T790M mutation status and clinical efficacy were compared between Asian and non‐Asian patients treated with Osimertinib.

To investigate the origins of heterogeneity, we conducted further subgroup analysis based on ethnicity to examine differences in the PFS in diverse populations. The study by some articles^13^, ^14^, ^15^, ^27^, ^28^, ^29^, ^30^, ^31^, ^32^ was carried out in a predominantly Asian population, while Goldberg et al.^26^ focused on a non‐Asian population. Subgroup analysis revealed that the HR of the T790M‐maintenance group versus the T790M‐loss group was comparable in the Asian and non‐Asian populations (HR: 0.46, 95% CI: 0.31–0.68 for Asians; HR: 0.12, 95% CI: 0.01–1.27 for non‐Asians; Figure 7). Because the 95% CI of the combined effect size of the two subgroups overlapped, the difference was not considered statistically significant.^36^ Thus, we held that there were no differences between Asian and non‐Asian NSCLC patients with respect to the impact of T790M mutation status on the clinical effects of Osimertinib resistance.

### 3.4 Publication bias

**FIGURE 8:**
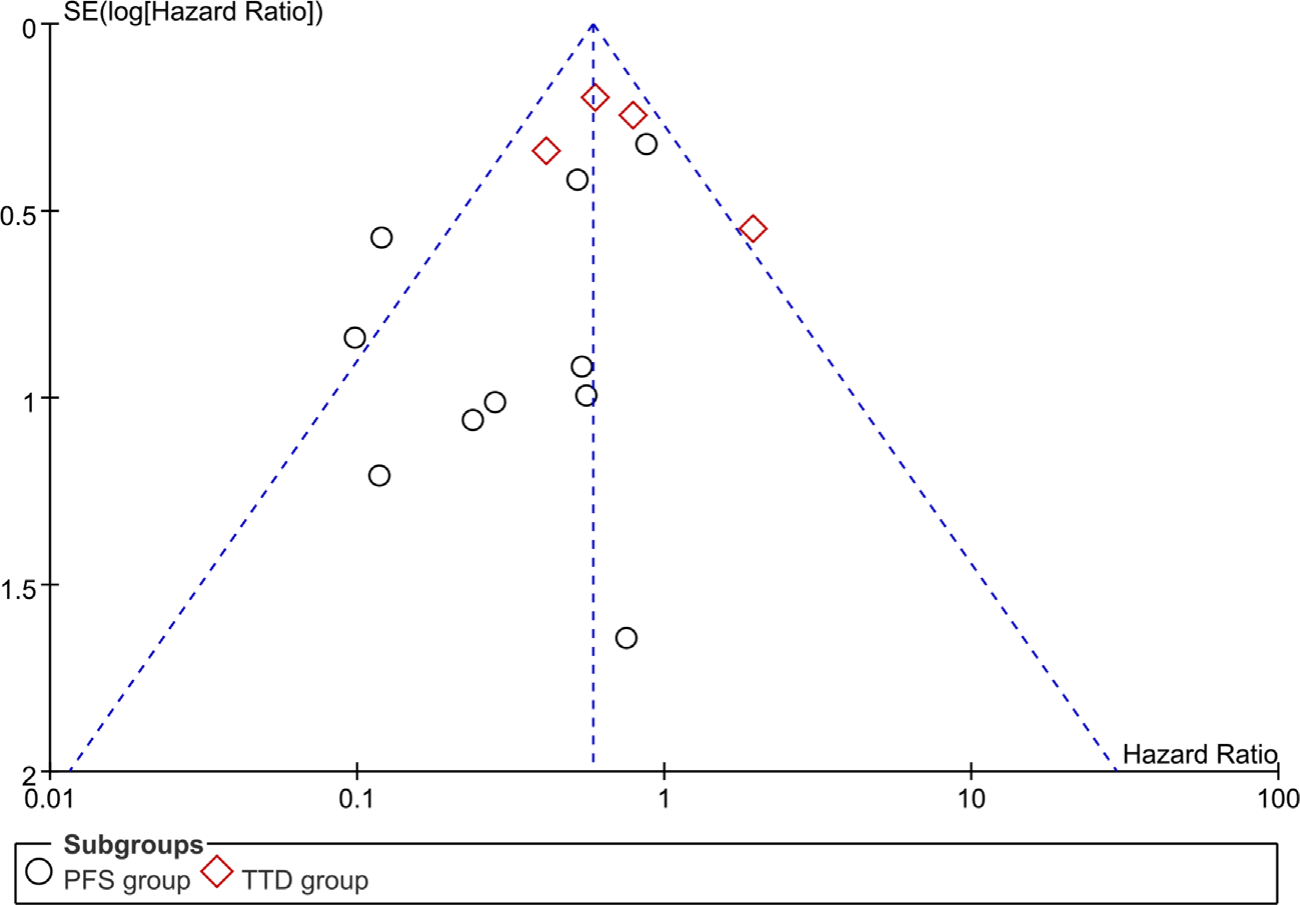
Funnel plots of the studies included in this meta‐analysis.

In the current study, we used Review Manager 5.4 software to generate funnel plots to evaluate bias. The funnel plot in Figure 8 has a missing Angle, indicating that there is publication bias in the included literature. However, because nine of the 14 papers are retrospective studies, the publication bias exists objectively with little heterogeneity. Therefore, we believe that publication bias did not affect the reliability of our results.

## 4 DISCUSSION

Our study aimed to assess the relationship between the clinical effectiveness of Osimertinib in NSCLC patients and the T790M mutation status following resistance to Osimertinib, as well as examine differences between plasma and tissue tests and between Asian and non‐Asian groups further. Here, we found that patients treated with Osimertinib had longer PFS, OS, and TTD and higher clinical benefits when the T790M mutation persisted after Osimertinib resistance. Compared with the blood test, the results of the tissue test are more significant. Furthermore, we found that both Asian and non‐Asian patients received similar clinical benefits.

NSCLC patients harboring EGFR mutations have a considerably longer survival rate associated with Osimertinib treatment. Osimertinib has been demonstrated to significantly increase the PFS and OS compared with treatment with pemetrexed plus platinum in the AURA3 study (NCT02151981),^37^ with a PFS of 10.1 months. The clinical efficacy and safety of Osimertinib in diverse populations have also been reported in the ASTRIS study (NCT02474355),^38^, ^39^, ^40^, ^41^, ^42^ which examined data from South Korea, Spain, Belgium, and other nations. The PFS and OS of the above studies were similar to the results of the articles included in the meta‐analysis.

However, with prolonged treatment duration, patients still face the inevitable drug resistance.^43^ The mechanisms underlying the second‐line Osimertinib resistance involve multiple genes with varying degrees of mutations.^11^, ^14^, ^44^ One study highlighted the need for EGFR T790M genotyping of tumor samples obtained after disease progression in patients treated with Osimertinib to guide treatment decisions.^45^ Effective targeted therapy requires timely detection of gene mutations therefore, it is crucial to identify the precise significant gene loci to test, predict, and even resolve Osimertinib resistance.^46^

Because the T790M mutation is a key target site of Osimertinib, the effectiveness of Osimertinib depends largely on whether the T790M mutation persists following treatment.^11^ According to recent studies,^14^, ^15^, ^28^, ^33^, ^47^, ^48^, ^49^ 34% to 75.9% of people had T790M gene deletions after developing Osimertinib resistance. As for the possible cause of T790M gene loss, most studies have suggested that it is mediated by the emergence of preexisting resistant clones^13^, ^33^, ^50^ because Osimertinib has selective pressure on the EGFR T790M gene, which may reverse the growth advantage of T790M‐positive and wild‐type cell clones. These cloned genes have additional EGFR‐independent resistance mechanisms that further contribute to the loss of the T790M gene upon resistance to Osimertinib.^32^ Consistent with this hypothesis, Zhao et al.^32^ found multiple EGFR‐independent resistance mechanisms in tumors lacking the T790M mutation, and Lee et al.^49^ found more bypass activation of independent EGFR in the T790M‐loss group than in the T790M‐maintenance group. However the prevalence of various non‐T790M resistance mechanisms shows that it may not be very realistic to evaluate the clinical effectiveness of Osimertinib based just on the status of the T790M mutation, and the coexistence of multiple resistance mechanisms is more clinically meaningful reality.^33^ Mu et al.^15^ found that patients with alternative pathway activation following Osimertinib resistance had shorter PFS and TD than patients with EGFR‐dependent resistance mechanisms (median PFS, 8.2 months vs. 13.5 months; median TD, 9.5 months vs. 16.6 months). Lee et al.^14^ suggested that this was exacerbated by MET amplification, which in patients with T790M gene loss had a significantly worse prognosis than those with other mutations, suggesting that prognosis depends not only on the loss of T790M itself but also on the nature or type of concomitant resistance alterations.

Additionally, the heterogeneity of the T790M assay, which comprised the heterogeneity of detection techniques and the heterogeneity of samples, also affects the detection accuracy.^51^, ^52^, ^53^ The majority of the included studies used tissue‐based or plasma‐based assays for genetic testing. Plasma‐based testing has many advantages over tissue biopsy, such as ease of use, but it can also produce some false positive results, which may affect the experimental results.^31^ Gray et al.^54^ conducted a recent study that demonstrated that the real‐time surveillance of circulating tumor DNA (ctDNA) in plasma has the potential to serve as a prognostic indicator for patient outcomes. EGFRm, or epidermal growth factor receptor mutation, encompasses various mutations such as exon 19 deletions or the L858R mutant within exon 21. The T790M mutation is primarily attributed to drug resistance induced by the patient's prior utilization of first‐ and second‐generation EGFR‐TKIs. The PFS of patients who exhibited plasma EGFRm clearance was found to be superior to that of patients who did not demonstrate EGFRm clearance, 3 weeks following treatment. However, the relationship between the dynamic changes in the T790M mutation and PFS of third‐generation TKIs is still unclear.^55^ Our findings indicate that the presence of the T790M mutation may enhance the therapeutic efficacy of Osimertinib. Compared with plasma samples, the results obtained from tissue samples are more pronounced. This could be attributed to the higher accuracy of tissue biopsy, which allows for a better reflection of the tumor tissue's condition. On the other hand, the T790M state of plasma may provide insights into the tumor load.^56^, ^57^, ^58^, ^59^ This highlights the need for future experiments to consider the variations caused by these two test methods.

Several previous studies have shown that quantitative assessment of the T790M mutation burden, rather than a binary status test, can better reflect the clinical response of third‐generation EGFR inhibitors.^33^, ^60^, ^61^ There is also research using genetic tests to see the genetic changes were observed in 0 months, 3 months, and 12 months and drug‐resistant, which confirmed that regular examination of the patient's genotype can better understand the patient's genetic changes and help to understand the mechanism of resistance to Osimertinib.^62^, ^63^ This indicates that the improvement of gene detection methods will continue to break the limitations of the current understanding of gene mutations. In the future, how to unify detection methods may reduce the error of test results and the error of combined results.

The effectiveness of Osimertinib has previously been shown to be significantly influenced by ethnicity.^64^ This may be due to the observed epidemiological and demographic variations of NSCLC patients from various racial backgrounds.^65^ Asians are more likely to have EGFR mutations, while South Asians are more likely to carry the drug‐resistant T790M mutation than people from other parts of the world.^66^ As a result, Asian populations are more likely than non‐Asian populations to use third‐generation EGFR‐TKIs that target the T790M mutation. Thus, the relative activities of various EGFR‐TKIs may vary between Asian and non‐Asian patients.^67^ In the current study, we carried out a subgroup analysis based on the ethnicity of the population and confirmed that Asian patients treated with Osimertinib had a similar HR to non‐Asian patients in the T790M‐maintenance group compared with the T790M‐loss group, indicating that the clinical benefits of Osimertinib in the Asian and non‐Asians population were higher both in terms of incidence probability and treatment effect, which are comparable to the findings of earlier investigations.^37^, ^38^, ^39^, ^40^, ^41^, ^49^, ^64^ However, in the current analysis, most of the studies in the Asian populations were single‐center trials carried out by Chinese and Korean research organizations, while only one study in non‐Asian populations were included. Thus, further studies are required to validate our findings.

Overall, the understanding of detailed genomic changes in tumors before and after Osimertinib treatment is not complete, and more clinical data are needed to reveal differences in T790M mutation status after Osimertinib treatment failure. In the current study, we performed a meta‐analysis study on data collected from the literature on drug resistance to Osimertinib from the past 10 years. Our findings were consistent with those of a previous meta‐analysis study.^13^ However, the previous meta‐analysis had some flaws, such as only analyzing eight studies up until 2019 and having a limited number of patients, including only 312 patients, which may have decreased the analysis's power. In addition, significant heterogeneity was observed in this meta‐analysis, which may undermine the robustness of the pooled analyses. However, compared with the previous report, our study included new studies from the past few years, as well as new indicators of PFS, OS, and TTD. In addition, we carried out two subgroup analyses to examine differences in clinical efficacy and T790M mutation status between plasma and tissue tests and between Asian and non‐Asian treated with Osimertinib. We addressed these concerns by increasing the sample size to 478 patients, reducing heterogeneity, and conducting subgroup analyses based on sample types and ethnicity. These enhancements allow the current study's findings to be more accurately generalized to a larger patient population and may make them a more trustworthy source of information for therapeutic decision‐making.

Our findings indicated that it is possible to predict the clinical benefits of Osimertinib treatment for NSCLC patients by regularly checking their T790M gene mutation status. This will help to better predict the effectiveness and drug resistance of Osimertinib and allow patients to undergo the most effective treatment regimens promptly. Conversely, for patients who have lost the T790M mutation, intensive treatment options, such as combining chemotherapy with EGFR‐TKI, may be considered to further enhance the therapeutic response. These inferences warrant further investigation. In addition, knowledge of the patient's T790M gene mutation status can also identify treatment history and determine possible mechanisms of acquired resistance to EGFR‐TKIs.^68^, ^69^

There are some limitations associated with our study. First, the studies included in our meta‐analysis were observational, and therefore some inherent biases cannot be avoided. In addition, most of the studies were based on Asian patients, primarily in China and South Korea; thus, the conclusions of our subgroup analysis need to be verified.

## 5 CONCLUSION

Taken together, our findings demonstrate that the persistence of the T790M mutation during Osimertinib treatment affects both the resistance and efficacy of Osimertinib, and compared with the blood tests, the results of the tissue tests are more significant. This study may support customized usage of Osimertinib and assist healthcare professionals in making clinical decisions.

## AUTHOR CONTRIBUTIONS

All authors contributed to the study conception and design. Conceptualization, data curation, formal analysis, investigation, writing–original draft preparation & review & editing were performed by Guo Liuxian. Data curation, formal analysis, writing–original draft preparation & review & editing were performed by Zhou Guojin. Conceptualization, funding acquisition, investigation, project administration, resources, writing–original draft preparation& review & editing were performed by Chen Jie. Conceptualization, funding acquisition, project administration, resources, writing–review & editing were performed by Huang Min, Tang Kejing, Xu Jing. All authors read and approved the final manuscript.

## CONFLICT OF INTEREST STATEMENT

The authors declare that there are no conflicts of interest.

## ETHICS STATEMENT

Not applicable.

## PUBLISHER'S NOTE

All claims expressed in this article are solely those of the authors and do not necessarily represent those of their affiliated organizations, or those of the publisher, the editors and the reviewers. Any product that may be evaluated in this article, or claim that may be made by its manufacturer, is not guaranteed or endorsed by the publisher.

## Supporting information


**Data S1.** Supporting Information.

## Data Availability

The original contributions presented in the study are included in the article/Supporting Information. Further inquiries can be directed to the corresponding author.
