# Supplementary material for: The impact of EGFR T790M mutation status following the development of Osimertinib resistance on the efficacy of Osimertinib in non‐small cell lung cancer: A meta‐analysis
Source: Clin Respir J. 2024 Apr 7;18(4):e13748. doi: 10.1111/crj.13748 (PMC10999367; doi:10.1111/crj.13748)
Supplement: Supplementary file 1 — Data S1. Supporting Information. [file CRJ-18-e13748-s001.docx]

**The impact of EGFR T790M mutation status following the development of Osimertinib resistance on the efficacy of Osimertinib in non-small cell lung cancer: a meta-analysis**

GUO Liuxian ^1,2^, ZHOU Guojin^1,2^, HUANG Min^3^, TANG Kejing^4^, XU Jing^5^, CHEN Jie^1*^

**CHEN Jie（Corresponding Author）:**

Department of Pharmacy, The First Affiliated Hospital, Sun Yat-sen University, 510080, Guangzhou, China.

Email：[Chenj28@mail.sysu.edu.cn](mailto:Chenj28@mail.sysu.edu.cn)

Tel：18902233518

**Search strategy:**

**PubMed** January 2014 to November 2023

("carcinoma, non small cell lung"[MeSH Terms] OR ("carcinoma"[All Fields] AND "non small cell"[All Fields] AND "lung"[All Fields]) OR "non-small-cell lung carcinoma"[All Fields] OR ("non"[All Fields] AND "small"[All Fields] AND "cell"[All Fields] AND "lung"[All Fields] AND "cancer"[All Fields]) OR "non small cell lung cancer"[All Fields] OR ("carcinoma, non small cell lung"[MeSH Terms] OR ("carcinoma"[All Fields] AND "non small cell"[All Fields] AND "lung"[All Fields]) OR "non-small-cell lung carcinoma"[All Fields] OR ("carcinoma"[All Fields] AND "non"[All Fields] AND "small"[All Fields] AND "cell"[All Fields] AND "lung"[All Fields]) OR "carcinoma non small cell lung"[All Fields]) OR ("carcinoma, non small cell lung"[MeSH Terms] OR ("carcinoma"[All Fields] AND "non small cell"[All Fields] AND "lung"[All Fields]) OR "non-small-cell lung carcinoma"[All Fields] OR ("carcinomas"[All Fields] AND "non"[All Fields] AND "small"[All Fields] AND "cell"[All Fields] AND "lung"[All Fields]) OR "carcinomas non small cell lung"[All Fields]) OR ("carcinoma, non small cell lung"[MeSH Terms] OR ("carcinoma"[All Fields] AND "non small cell"[All Fields] AND "lung"[All Fields]) OR "non-small-cell lung carcinoma"[All Fields] OR ("lung"[All Fields] AND "carcinoma"[All Fields] AND "non"[All Fields] AND "small"[All Fields] AND "cell"[All Fields]) OR "lung carcinoma non small cell"[All Fields]) OR ("carcinoma, non small cell lung"[MeSH Terms] OR ("carcinoma"[All Fields] AND "non small cell"[All Fields] AND "lung"[All Fields]) OR "non-small-cell lung carcinoma"[All Fields] OR ("lung"[All Fields] AND "carcinomas"[All Fields] AND "non"[All Fields] AND "small"[All Fields] AND "cell"[All Fields]) OR "lung carcinomas non small cell"[All Fields]) OR ("carcinoma, non small cell lung"[MeSH Terms] OR ("carcinoma"[All Fields] AND "non small cell"[All Fields] AND "lung"[All Fields]) OR "non-small-cell lung carcinoma"[All Fields] OR ("non"[All Fields] AND "small"[All Fields] AND "cell"[All Fields] AND "lung"[All Fields] AND "carcinomas"[All Fields]) OR "non small cell lung carcinomas"[All Fields]) OR ("carcinoma, non small cell lung"[MeSH Terms] OR ("carcinoma"[All Fields] AND "non small cell"[All Fields] AND "lung"[All Fields]) OR "non-small-cell lung carcinoma"[All Fields] OR ("non"[All Fields] AND "small"[All Fields] AND "cell"[All Fields] AND "lung"[All Fields] AND "carcinoma"[All Fields]) OR "non-small-cell lung carcinoma"[All Fields]) OR ("carcinoma, non small cell lung"[MeSH Terms] OR ("carcinoma"[All Fields] AND "non small cell"[All Fields] AND "lung"[All Fields]) OR "non-small-cell lung carcinoma"[All Fields] OR ("non"[All Fields] AND "small"[All Fields] AND "cell"[All Fields] AND "lung"[All Fields] AND "carcinoma"[All Fields]) OR "non-small-cell lung carcinoma"[All Fields]) OR ("carcinoma, non small cell lung"[MeSH Terms] OR ("carcinoma"[All Fields] AND "non small cell"[All Fields] AND "lung"[All Fields]) OR "non-small-cell lung carcinoma"[All Fields] OR ("carcinoma"[All Fields] AND "non"[All Fields] AND "small"[All Fields] AND "cell"[All Fields] AND "lung"[All Fields]) OR "carcinoma non small cell lung"[All Fields]) OR ("carcinoma, non small cell lung"[MeSH Terms] OR ("carcinoma"[All Fields] AND "non small cell"[All Fields] AND "lung"[All Fields]) OR "non-small-cell lung carcinoma"[All Fields] OR ("non"[All Fields] AND "small"[All Fields] AND "cell"[All Fields] AND "lung"[All Fields] AND "carcinoma"[All Fields]) OR "non-small-cell lung carcinoma"[All Fields]) OR ("carcinoma, non small cell lung"[MeSH Terms] OR ("carcinoma"[All Fields] AND "non small cell"[All Fields] AND "lung"[All Fields]) OR "non-small-cell lung carcinoma"[All Fields] OR ("non"[All Fields] AND "small"[All Fields] AND "cell"[All Fields] AND "lung"[All Fields] AND "cancer"[All Fields]) OR "non small cell lung cancer"[All Fields]) OR ("carcinoma, non small cell lung"[MeSH Terms] OR ("carcinoma"[All Fields] AND "non small cell"[All Fields] AND "lung"[All Fields]) OR "non-small-cell lung carcinoma"[All Fields] OR ("nonsmall"[All Fields] AND "cell"[All Fields] AND "lung"[All Fields] AND "cancer"[All Fields]) OR "nonsmall cell lung cancer"[All Fields]) OR "Adenocarcinoma"[MeSH Terms] OR "Adenocarcinoma"[All Fields] OR "Adenocarcinomas"[All Fields] OR "adenocarcinoma s"[All Fields] OR "Adenocarcinomas"[Title/Abstract] OR "adenoma malignant"[Title/Abstract] OR "adenomas malignant"[Title/Abstract] OR "malignant adenoma"[Title/Abstract] OR "malignant adenomas"[Title/Abstract] OR "carcinoma granular cell"[Title/Abstract] OR "carcinomas granular cell"[Title/Abstract] OR "granular cell carcinoma"[Title/Abstract] OR "granular cell carcinomas"[Title/Abstract] OR "adenocarcinoma granular cell"[Title/Abstract] OR (("Adenocarcinoma"[MeSH Terms] OR "Adenocarcinoma"[All Fields] OR "Adenocarcinomas"[All Fields] OR "adenocarcinoma s"[All Fields]) AND "granular cell"[Title/Abstract]) OR "granular cell adenocarcinoma"[Title/Abstract] OR (("Granular"[All Fields] OR "granulars"[All Fields]) AND "cell adenocarcinomas"[Title/Abstract]) OR "adenocarcinoma tubular"[Title/Abstract] OR "adenocarcinomas tubular"[Title/Abstract] OR "tubular adenocarcinoma"[Title/Abstract] OR "tubular adenocarcinomas"[Title/Abstract] OR "carcinoma tubular"[Title/Abstract] OR "carcinomas tubular"[Title/Abstract] OR "tubular carcinoma"[Title/Abstract] OR "tubular carcinomas"[Title/Abstract] OR "carcinoma cribriform"[Title/Abstract] OR "carcinomas cribriform"[Title/Abstract] OR "cribriform carcinoma"[Title/Abstract] OR "cribriform carcinomas"[Title/Abstract] OR "adenocarcinoma basal cell"[Title/Abstract] OR "adenocarcinomas basal cell"[Title/Abstract] OR "basal cell adenocarcinoma"[Title/Abstract] OR "basal cell adenocarcinomas"[Title/Abstract] OR (("Adenocarcinoma"[MeSH Terms] OR "Adenocarcinoma"[All Fields] OR "Adenocarcinomas"[All Fields] OR "adenocarcinoma s"[All Fields]) AND "Oxyphilic"[Title/Abstract]) OR (("Adenocarcinoma"[MeSH Terms] OR "Adenocarcinoma"[All Fields] OR "Adenocarcinomas"[All Fields] OR "adenocarcinoma s"[All Fields]) AND "Oxyphilic"[Title/Abstract]) OR "oxyphilic adenocarcinoma"[Title/Abstract] OR (("oxyphil"[All Fields] OR "Oxyphilic"[All Fields]) AND "Adenocarcinomas"[Title/Abstract])) AND "T790M"[All Fields] AND ("egfr protein human"[Supplementary Concept] OR "egfr protein human"[All Fields] OR "egfr protein human"[All Fields] OR ("egfr protein human"[Supplementary Concept] OR "egfr protein human"[All Fields]) OR ("egfr protein human"[Supplementary Concept] OR "egfr protein human"[All Fields]) OR ("egfr protein human"[Supplementary Concept] OR "egfr protein human"[All Fields]) OR ("egfr protein human"[Supplementary Concept] OR "egfr protein human"[All Fields]) OR (("erbb receptors"[MeSH Terms] OR ("erbb"[All Fields] AND "receptors"[All Fields]) OR "erbb receptors"[All Fields] OR ("epidermal"[All Fields] AND "growth"[All Fields] AND "factor"[All Fields] AND "receptor"[All Fields]) OR "epidermal growth factor receptor"[All Fields]) AND (("leukemia, erythroblastic, acute"[MeSH Terms] OR ("leukemia"[All Fields] AND "erythroblastic"[All Fields] AND "acute"[All Fields]) OR "acute erythroblastic leukemia"[All Fields] OR ("erythroblastic"[All Fields] AND "leukemia"[All Fields]) OR "erythroblastic leukemia"[All Fields]) AND ("virally"[All Fields] OR "virals"[All Fields] OR "virology"[MeSH Terms] OR "virology"[All Fields] OR "viral"[All Fields]) AND "v-erb-b"[All Fields] AND (("carcinogens"[Pharmacological Action] OR "carcinogens"[MeSH Terms] OR "carcinogens"[All Fields] OR "oncogen"[All Fields] OR "oncogens"[All Fields] OR "oncogenes"[MeSH Terms] OR "oncogenes"[All Fields] OR "oncogene"[All Fields] OR "oncogenic"[All Fields] OR "oncogenically"[All Fields] OR "oncogenicities"[All Fields] OR "oncogenicity"[All Fields]) AND ("homolog"[All Fields] OR "homologous"[All Fields] OR "homologously"[All Fields] OR "homologs"[All Fields]) AND ("birds"[MeSH Terms] OR "birds"[All Fields] OR "avian"[All Fields] OR "avians"[All Fields]))) AND (("protein s"[All Fields] OR "proteinous"[All Fields] OR "proteins"[MeSH Terms] OR "proteins"[All Fields] OR "protein"[All Fields]) AND ("human s"[All Fields] OR "humans"[MeSH Terms] OR "humans"[All Fields] OR "human"[All Fields])))) AND ("osimertinib"[Supplementary Concept] OR "osimertinib"[All Fields] OR ("n"[All Fields] AND ("2"[All Fields] AND ("2"[All Fields] AND "dimethylamino"[All Fields] AND ("ethyl"[All Fields] OR "ethylate"[All Fields] OR "ethylated"[All Fields] OR "ethylates"[All Fields] OR "ethylating"[All Fields] OR "ethylation"[All Fields] OR "ethylations"[All Fields] OR "ethyls"[All Fields]) AND "methylamino"[All Fields]) AND "4 methoxy 5"[All Fields] AND ("4"[All Fields] AND "1-methyl-1H-indol-3-yl"[All Fields] AND "2 pyrimidinyl"[All Fields] AND ("amino"[All Fields] OR "aminos"[All Fields])) AND ("phenyl"[All Fields] OR "phenylated"[All Fields] OR "phenylation"[All Fields] OR "phenylic"[All Fields] OR "phenyls"[All Fields])) AND ("acrylamide"[MeSH Terms] OR "acrylamide"[All Fields] OR "2 propenamide"[All Fields])) OR ("osimertinib"[Supplementary Concept] OR "osimertinib"[All Fields] OR "mereletinib"[All Fields]) OR ("osimertinib"[Supplementary Concept] OR "osimertinib"[All Fields] OR "osimertinib mesylate"[All Fields]) OR ("osimertinib"[Supplementary Concept] OR "osimertinib"[All Fields]) OR ("osimertinib"[Supplementary Concept] OR "osimertinib"[All Fields]) OR ("n"[All Fields] AND ("2"[All Fields] AND ("2"[All Fields] AND "dimethylamino"[All Fields] AND ("ethyl"[All Fields] OR "ethylate"[All Fields] OR "ethylated"[All Fields] OR "ethylates"[All Fields] OR "ethylating"[All Fields] OR "ethylation"[All Fields] OR "ethylations"[All Fields] OR "ethyls"[All Fields]) AND "methylamino"[All Fields]) AND "4 methoxy 5"[All Fields] AND ("4"[All Fields] AND "1-methyl-1H-indol-3-yl"[All Fields] AND "2 pyrimidinyl"[All Fields] AND ("amino"[All Fields] OR "aminos"[All Fields])) AND ("phenyl"[All Fields] OR "phenylated"[All Fields] OR "phenylation"[All Fields] OR "phenylic"[All Fields] OR "phenyls"[All Fields])) AND (("acrylamide"[MeSH Terms] OR "acrylamide"[All Fields] OR "2 propenamide"[All Fields]) AND ("mesyl"[All Fields] OR "mesylated"[All Fields] OR "mesylates"[MeSH Terms] OR "mesylates"[All Fields] OR "mesilate"[All Fields] OR "mesylate"[All Fields] OR "methanesulfonates"[All Fields] OR "mesylation"[All Fields] OR "methanesulfonic acid"[Supplementary Concept] OR "methanesulfonic acid"[All Fields] OR "methanesulfonate"[All Fields] OR "methanesulphonate"[All Fields] OR "methanesulphonates"[All Fields])) AND "1 1"[All Fields]) OR ("osimertinib"[Supplementary Concept] OR "osimertinib"[All Fields]) OR ("osimertinib"[Supplementary Concept] OR "osimertinib"[All Fields]) OR ("osimertinib"[Supplementary Concept] OR "osimertinib"[All Fields]) OR ("osimertinib"[Supplementary Concept] OR "osimertinib"[All Fields] OR "azd9291"[All Fields]) OR ("osimertinib"[Supplementary Concept] OR "osimertinib"[All Fields] OR "azd 9291"[All Fields]) OR ("osimertinib"[Supplementary Concept] OR "osimertinib"[All Fields] OR "tagrisso"[All Fields])) AND ("drug resistance, neoplasm"[MeSH Terms] OR ("drug"[All Fields] AND "resistance"[All Fields] AND "neoplasm"[All Fields]) OR "neoplasm drug resistance"[All Fields] OR ("drug"[All Fields] AND "resistance"[All Fields] AND "neoplasm"[All Fields]) OR "drug resistance neoplasm"[All Fields] OR ("drug resistance, neoplasm"[MeSH Terms] OR ("drug"[All Fields] AND "resistance"[All Fields] AND "neoplasm"[All Fields]) OR "neoplasm drug resistance"[All Fields] OR ("resistance"[All Fields] AND "antineoplastic"[All Fields] AND "agent"[All Fields])) OR ("drug resistance, neoplasm"[MeSH Terms] OR ("drug"[All Fields] AND "resistance"[All Fields] AND "neoplasm"[All Fields]) OR "neoplasm drug resistance"[All Fields] OR ("resistance"[All Fields] AND "antineoplastic"[All Fields] AND "drug"[All Fields])) OR ("drug resistance, neoplasm"[MeSH Terms] OR ("drug"[All Fields] AND "resistance"[All Fields] AND "neoplasm"[All Fields]) OR "neoplasm drug resistance"[All Fields] OR ("drug"[All Fields] AND "resistance"[All Fields] AND "antineoplastic"[All Fields]) OR "drug resistance antineoplastic"[All Fields]) OR ("drug resistance, neoplasm"[MeSH Terms] OR ("drug"[All Fields] AND "resistance"[All Fields] AND "neoplasm"[All Fields]) OR "neoplasm drug resistance"[All Fields] OR ("neoplasm"[All Fields] AND "drug"[All Fields] AND "resistance"[All Fields])) OR ("drug resistance, neoplasm"[MeSH Terms] OR ("drug"[All Fields] AND "resistance"[All Fields] AND "neoplasm"[All Fields]) OR "neoplasm drug resistance"[All Fields] OR ("antineoplastic"[All Fields] AND "agent"[All Fields] AND "resistance"[All Fields]) OR "antineoplastic agent resistance"[All Fields]) OR ("drug resistance, neoplasm"[MeSH Terms] OR ("drug"[All Fields] AND "resistance"[All Fields] AND "neoplasm"[All Fields]) OR "neoplasm drug resistance"[All Fields] OR ("antineoplastic"[All Fields] AND "drug"[All Fields] AND "resistance"[All Fields]) OR "antineoplastic drug resistance"[All Fields]) OR ("drug resistance, neoplasm"[MeSH Terms] OR ("drug"[All Fields] AND "resistance"[All Fields] AND "neoplasm"[All Fields]) OR "neoplasm drug resistance"[All Fields] OR ("antibiotic"[All Fields] AND "resistance"[All Fields] AND "neoplasm"[All Fields])))

| **#** | **Searches** | **Results** |
| --- | --- | --- |
| 1 | Non-small cell lung cancer | 106183 |
| 2 | T790M | 3069 |
| 3 | EGFR | 14189 |
| 4 | Osimertinib | 2786 |
| 5 | Drug Resistance, Neoplasm | 97835 |
| 6 | Adenocarcinoma | 521039 |
| 7 | (1 OR 6) AND 2 AND 3 AND 4 AND 5 | 249 |

**Embase** January 2014 to November 2023

1.'non small cell lung cancer' OR (carcinomas, AND 'non small cell' AND lung) OR (lung AND carcinoma, AND 'non small cell') OR (lung AND carcinomas, AND 'non small cell') OR ('non small cell' AND lung AND carcinomas) OR ('non small cell' AND lung AND carcinoma) OR (non AND small AND cell AND lung AND carcinoma) OR (carcinoma, AND 'non small' AND cell AND lung) OR ('non small' AND cell AND lung AND carcinoma) OR ('non small' AND cell AND lung AND cancer) OR (nonsmall AND cell AND lung AND cancer)

2.T790M

3.(errp OR erbb OR egfr) OR (epidermal AND growth AND factor AND receptor AND related) OR her1 OR (epidermal AND growth AND factor AND receptor AND erythroblastic AND leukemia AND viral AND 'v erb b' AND oncogene AND homolog, AND avian) OR (errp AND protein, AND human) OR (erbb AND protein, AND human) OR (egfr AND protein, AND human) OR (epidermal AND growth AND factor AND receptor AND related AND protein, AND human) OR (her1 AND protein, AND human) OR (epidermal AND growth AND factor AND receptor AND erythroblastic AND leukemia AND viral AND 'v erb b' AND oncogene AND homolog, AND avian AND protein, AND human)

4.osimertinib OR mereletinib OR (osimertinib AND mesylate) OR (osimertinib AND mesilate) OR (mereletinib AND mesilate) OR (azd9291 AND mesylate) OR (mereletinib AND mesylate) OR ('azd 9291' AND mesylate) OR azd9291 OR 'azd 9291' OR tagrisso

5.drug AND resistance, AND neoplasm OR (resistance, AND antineoplastic AND agent) OR (resistance, AND antineoplastic AND drug) OR (drug AND resistance, AND antineoplastic) OR (neoplasm AND drug AND resistance) OR (antineoplastic AND agent AND resistance) OR (antineoplastic AND drug AND resistance) OR (antibiotic AND resistance, AND neoplasm)

6.adenocarcinoma OR adenocarcinomas OR (adenoma AND malignant) OR (adenomas AND malignant) OR (malignant AND adenoma) OR (malignant AND adenomas) OR (carcinoma AND granular cell) OR (carcinomas AND granular cell) OR (granular AND cell AND carcinoma) OR (granular AND cell AND carcinomas) OR (adenocarcinoma AND granular AND cell) OR (adenocarcinomas AND granular AND cell) OR (granular AND cell AND adenocarcinoma) OR (granular AND cell AND adenocarcinomas) OR (adenocarcinoma AND tubular) OR (adenocarcinomas AND tubular) OR (tubular AND adenocarcinomas) OR (tubular AND adenocarcinoma) OR (carcinoma AND tubular) OR (carcinomas AND tubular) OR (tubular AND carcinoma) OR (tubular AND carcinomas) OR (carcinoma AND cribriform) OR (carcinomas AND cribriform) OR (cribriform AND carcinoma) OR (cribriform AND carcinomas) OR (adenocarcinoma AND basal AND cell) OR (adenocarcinomas AND basal AND cell) OR (basal AND cell AND adenocarcinoma) OR (basal AND cell AND adenocarcinomas) OR (adenocarcinoma AND oxyphilic) OR (adenocarcinomas AND oxyphilic) OR (oxyphilic AND adenocarcinoma) OR (oxyphilic AND adenocarcinomas)

| **#** | **Searches** | **Results** |
| --- | --- | --- |
| 1 | Non-small cell lung cancer | 237585 |
| 2 | T790M | 6483 |
| 3 | EGFR | 190260 |
| 4 | Osimertinib | 8165 |
| 5 | Drug Resistance, Neoplasm | 245511 |
| 6 | Adenocarcinoma | 459927 |
| 7 | (1 OR 6) AND 2 AND 3 AND 4 AND 5 | 1531 |

**Web of science** January 2014 to November 2023

1.TS=(non-small cell lung cancer) OR TS=(Carcinoma, Non Small Cell Lung) OR TS=(Carcinomas, Non-Small-Cell Lung) OR TS=(Lung Carcinoma, Non-Small-Cell) OR TS=(Lung Carcinomas, Non-Small-Cell) OR TS=(Non-Small-Cell Lung Carcinomas) OR TS=(Non-Small-Cell Lung Carcinoma) OR TS=(Non Small Cell Lung Carcinoma) OR TS=(Carcinoma, Non-Small Cell Lung) OR TS=(Non-Small Cell Lung Carcinoma) OR TS=(Non-Small Cell Lung Cancer) OR TS=(Nonsmall Cell Lung Cancer)

2.TS=(T790M)

3.TS=(EGFR protein, human) OR TS=(ERRP protein, human) OR TS=(ERBB protein, human) OR TS=(epidermal growth factor receptor related protein, human) OR TS=(HER1 protein, human) OR TS=(epidermal growth factor receptor (erythroblastic leukemia viral (v-erb-b) oncogene homolog, avian protein, human)) OR TS=(EGFR) OR TS=(ERRP) OR TS=(ERBB) OR TS=(epidermal growth factor receptor related) OR TS=(HER1) OR TS=(epidermal growth factor receptor (erythroblastic leukemia viral (v-erb-b) oncogene homolog, avian))

4.TS=(Osimertinib) OR TS=(N-(2-((2-(dimethylamino)ethyl)methylamino)-4-methoxy-5-((4-(1-methyl-1H-indol-3-yl)-2-pyrimidinyl)amino)phenyl)-2-propenamide) OR TS=(mereletinib) OR TS=(osimertinib mesylate) OR TS=(osimertinib mesilate) OR TS=(mereletinib mesilate) OR TS=(N-(2-((2-(dimethylamino)ethyl)methylamino)-4-methoxy-5-((4-(1-methyl-1H-indol-3-yl)-2-pyrimidinyl)amino)phenyl)-2-propenamide methanesulfonate (1:1)) OR TS=(AZD9291 mesylate) OR TS=(mereletinib mesylate) OR TS=(AZD-9291 mesylate) OR TS=(AZD9291) OR TS=(AZD-9291) OR TS=(Tagrisso)

5.TS=(Drug Resistance, Neoplasm) OR TS=(Resistance, Antineoplastic Agent) OR TS=(Resistance, Antineoplastic Drug) OR TS=(Drug Resistance, Antineoplastic) OR TS=(Neoplasm Drug Resistance) OR TS=(Antineoplastic Agent Resistance) OR TS=(Antineoplastic Drug Resistance) OR TS=(Antibiotic Resistance, Neoplasm)

6.(TS=(Adenocarcinoma) OR TS=(Adenocarcinomas) OR TS=(Adenoma, Malignant)) OR TS=(Adenomas, Malignant)) OR TS=(Malignant Adenoma)) OR TS=(Malignant Adenomas)) OR TS=(Carcinoma, Granular Cell)) OR TS=(Carcinomas, Granular Cell)) OR TS=(Granular Cell Carcinoma)) OR TS=(Granular Cell Carcinomas)) OR TS=(Adenocarcinoma, Granular Cell)) OR TS=(Adenocarcinomas, Granular Cell)) OR TS=(Granular Cell Adenocarcinoma)) OR TS=(Granular Cell Adenocarcinomas)) OR TS=(Adenocarcinoma, Tubular)) OR TS=(Adenocarcinomas, Tubular)) OR TS=(Tubular Adenocarcinoma)) OR TS=(Tubular Adenocarcinomas)) OR TS=(Carcinoma, Tubular)) OR TS=(Carcinomas, Tubular)) OR TS=(Tubular Carcinoma)) OR TS=(Tubular Carcinomas)) OR TS=(Carcinoma, Cribriform)) OR TS=(Carcinomas, Cribriform)) OR TS=(Cribriform Carcinoma)) OR TS=(Cribriform Carcinomas)) OR TS=(Adenocarcinoma, Basal Cell)) OR TS=(Adenocarcinomas, Basal Cell)) OR TS=(Basal Cell Adenocarcinoma)) OR TS=(Basal Cell Adenocarcinomas)) OR TS=(Adenocarcinoma, Oxyphilic)) OR TS=(Adenocarcinomas, Oxyphilic)) OR TS=(Oxyphilic Adenocarcinoma)) OR TS=(Oxyphilic Adenocarcinomas)

| **#** | **Searches** | **Results** |
| --- | --- | --- |
| 1 | non-small cell lung cancer | 190526 |
| 2 | T790M | 4866 |
| 3 | EGFR | 190882 |
| 4 | Osimertinib | 5329 |
| 5 | Drug Resistance, Neoplasm | 249006 |
| 6 | Adenocarcinoma | 504573 |
| 7 | (1 OR 6) AND 2 AND 3 AND 4 AND 5 | 861 |

The Searchable link of Web of science:

[https://www.webofscience.com/wos/alldb/summary/c803e0f4-c1a7-45bb-aaab-4d997e4fa2f5-9d6e9554/relevance/1](https://www.webofscience.com/wos/alldb/summary/e64fec85-8990-428c-9d50-88b1f311c3e7-7f623d61/relevance/1)

**Cochrane** January 2014 to November 2023

1.MeSH descriptor: [Carcinoma, Non-Smal-Cell Lung] explode all trees

2.(non-small cell lung cancer) (Word variations have been searched)

3.(T790M) (Word variations have been searched)

4.(EGFR) (Word variations have been searched)

5.MeSH descriptor: [Genes, erbB-1] explode all trees

6.(Osimertinib) (Word variations have been searched)

7.(Tagrisso) (Word variations have been searched)

8.(AZD9291)(Word variations have been searched)

9.MeSH descriptor: [Drug Resistance, Neoplasm] explode all trees

10.(Drug Resistance) (Word variations have been searched)

11.(Adenocarcinoma)(Word variations have been searched)

12.MeSH descriptor: [Adenocarcinoma] explode all trees

| **#** | **Searches** | **Results** |
| --- | --- | --- |
| 1 | MeSH descriptor: [Carcinoma, Non-Smal-Cell Lung] explode all trees | 5860 |
| 2 | (non-small cell lung cancer) (Word variations have been searched) | 15782 |
| 3 | (T790M) (Word variations have been searched) | 391 |
| 4 | (EGFR) (Word variations have been searched) | 12735 |
| 5 | MeSH descriptor: [Genes, erbB-1] explode all trees | 37 |
| 6 | (Osimertinib) (Word variations have been searched) | 423 |
| 7 | (Tagrisso) (Word variations have been searched) | 23 |
| 8 | (AZD9291)(Word variations have been searched) | 47 |
| 9 | MeSH descriptor: [Drug Resistance, Neoplasm] explode all trees | 884 |
| 10 | (Drug Resistance) (Word variations have been searched) | 37369 |
| 11 | (Adenocarcinoma)(Word variations have been searched) | 13394 |
| 12 | MeSH descriptor: [Adenocarcinoma] explode all trees | 10489 |
| 11 | (1 OR 2 OR 11 OR 12) AND 3 AND (4 OR 5) AND (6 OR 7 OR 8) AND (9 OR 10) | 86 |
